# Cancer screening rate in people with diabetes in the Korean population: results from the Korea National Health and Nutrition Examination Survey 2007-2009

**DOI:** 10.4178/epih.e2017036

**Published:** 2017-08-10

**Authors:** Kumban Walter Chuck, Minji Hwang, Kui Son Choi, Mina Suh, Jae Kwan Jun, Boyoung Park

**Affiliations:** 1 Department of Cancer Control and Population Health, National Cancer Center Graduate School of Cancer Science and Policy, Goyang, Korea; 2 Research Center, National Cancer Center, Goyang, Korea; 3 National Cancer Control Institute, National Cancer Center, Goyang, Korea

**Keywords:** Early detection of cancer, Diabetes mellitus, Nationwide cross-sectional study, Korea National Health and Nutrition Examination Survey

## Abstract

**OBJECTIVES:**

To investigate the screening rates for gastric, breast, and cervical cancer in people with diabetes compared with people without diabetes.

**METHODS:**

Data from the Korea National Health and Nutrition Examination Survey (2007-2009) were used. Cancer-free men who were 40 years old and over and cancer-free women who were 30 years old and over were included. The lifetime screening rate and regular screening rate were compared in people with and without diabetes.

**RESULTS:**

Fewer people with diabetes than people without diabetes had ever received cancer screening (53.5 vs. 59.5%, p<0.001 for gastric cancer; 60.5 vs. 71.5%, p<0.001 for breast cancer; and 49.1 vs. 59.6%, p<0.001 for cervical cancer). Fewer people with diabetes than people without diabetes received the recommended screenings for gastric cancer (38.9 vs. 42.9%, p<0.001), breast cancer (38.8 vs. 44.6%, p<0.001), and cervical cancer (35.1 vs. 51.2%, p<0.001). In subgroup analyses according to socioeconomic factors, the lifetime and recommended screening rates were lower in the diabetic population in most socioeconomic subgroups. In the multivariate analysis adjusted for socioeconomic factors, people with diabetes showed lower lifetime screening rates for gastric and cervical cancer (odds ratio [OR], 0.8; 95% confidence interval [CI], 0.7 to 0.9 and OR, 0.7; 95% CI, 0.6 to 0.9), and lower regular screening rates for breast and cervical cancer (OR, 0.7; 95% CI, 0.6 to 0.9 and OR, 0.7; 95% CI, 0.5 to 0.9).

**CONCLUSIONS:**

The cancer screening rate in people with diabetes was lower than in people without diabetes. Considering the higher cancer risk in people with diabetes, efforts to increase the screening rate in this high-risk population should be implemented.

## INTRODUCTION

Cancer is one of the major public health burdens in both developed and developing countries; it leads to increased mortality, and its incidence is increasing [1]. The global burden of diabetes has nearly doubled since 1980, from 4.7 to 8.5% in the adult population. This reflects an increase in associated risk factors such as obesity [2]. Diabetes and cancer are both chronic diseases that cause a health burden worldwide, and some epidemiologic studies have suggested that individuals with diabetes have a significantly higher risk for cancer, including gastric, breast, and cervical cancers [3-6]. Increasing the screening rates for these cancers in people with diabetes will go a long way to reduce the burden related to the cancer-related sequelae of diabetes [7]. Thus, several studies have examined the cancer screening rate in people with diabetes, but have shown some discrepancies in the cancer screening rates of people with diabetes compared to people without diabetes [8-10].

In Korea, cancer has been one of the leading causes of death since 1983 [11], and is also expected to become more common due to aging and westernized lifestyles [12]. In 1999, the National Cancer Screening Program (NCSP) was implemented by the Korean government as an organized cancer screening program, and opportunistic screenings are also provided depending on individuals’ needs [13]. Diabetes and its complications have also emerged as a major cause of morbidity and mortality. The prevalence of diabetes has increased 6-7-fold in the past 40 years [14]; although diabetes-related deaths have decreased since 2002, it still remains the fifth most common cause of death in Korea [15].

To the best of the authors’ knowledge, few studies in Korea have been conducted to assess whether people with diabetes receive less, more, or equal cancer screening in comparison to those without diabetes. Therefore, this study aimed to investigate the screening rates for gastric, breast, and cervical cancer, which are included in the NCSP [13], in people with diabetes in comparison with people without diabetes.

## MATERIALS AND METHODS

The primary source of data for this study was the Korea National Health and Nutrition Examination Survey (KNHANES) from 2007 to 2009. This is a nationwide survey that was put in place in 1998, and was initially conducted on a triennial basis. Since 2007, the survey has been conducted annually by the Korea Centers for Disease Control and Prevention to evaluate the health and nutritional conditions of the Korean population. The measurements in the KNHANES include direct physical examinations, clinical laboratory examinations, and personal interviews. The diabetic population was defined as including (1) people who had been diagnosed with diabetes by a physician and had received treatment for diabetes with insulin or medication, and (2) those with a fasting glucose level ≥ 126 mg/dL. The non-diabetic population included people who did not meet these criteria: that is, those who had never been diagnosed with diabetes by a physician, those who had never received treatment for diabetes with insulin or medication, and those with a fasting glucose level < 126 mg/dL.

In Korea, an organized cancer screening program is available for men and women in the general population 40 years of age and above, men and women 50 years of age and above, women 40 years of age and above, and women 30 years of age and above for gastric, colorectal, breast, and cervical cancer, respectively, and participants in each age category were included in the analysis of each type of cancer screening. For colorectal cancer, the option in the questionnaire that was used to estimate the most recent screening was different (within 2 years) from the period recommended by the NCSP (1-year interval), so we excluded the colorectal cancer screening rate from the analysis.

Information about cancer screening for these 4 cancers was collected by questionnaire from study participants, as well as sociodemographic characteristics. Income was analyzed based on monthly household income as follows: lowest (< 1.0 million Korean won [KRW] per month), lower (1.0-2.5 million KRW per month), higher (2.5-4.0 million KRW per month), and highest (> 4.0 million KRW per month) (note, 1,000 KRW= 1 US dollar). Educational level was classified as follows: elementary, middle school, high school, and college. Participants were classified by residential area based on cities and districts, which include multiple ‘dong’, and counties, which include multiple ‘eup’ and ‘myeon’. In this study, we classified dong as urban areas and ‘eup’ and ‘myeon’ as rural areas. Medical insurance was divided into the National Health Insurance (NHI) program, which is compulsory by law for all residing in the territory of Korea, and the Medical Aid program put in place by the government to ensure minimum living standards for low-income households. The questions that were analyzed included “Have you ever undergone gastric, breast, or cervical cancer screening?” If yes, participants were asked “Which screening method was used, and when was the last time that you underwent screening?” The cancer screening rate for each cancer was estimated in terms of ever-screened people, including those who had received a single screening in their lifetime, and people who had regular screening according to the guidelines recommended by NCSP, which are shown in Appendix 1. For gastric, breast, and cervical cancer screening, subjects who had undergone screening within the past 2 years were considered as having been regularly screened.

The baseline characteristics of people with and without diabetes were calculated using summary statistics and percentages and compared using the chi-square test. The weighted frequencies and screening rate percentages were calculated for the 4 different cancers at 95% confidence intervals (CIs), and we compared the screening rates between people with and without diabetes. Multivariate logistic regression was done for gastric, breast, and cervical cancer screening in diabetic and non-diabetic participants after adjusting for age, gender, income, education, residential area, and medical insurance. Stata version 12.0 (StataCorp., College Station, TX, USA) was used for all analyses.

## RESULTS

This study involved a total of 13,694 participants, including 1,500 with diabetes and 12,194 without diabetes. For gastric cancer, 10,554 participants were analyzed, including 1,436 with diabetes and 9,118 without diabetes. For breast cancer screening, 6,069 women participants, including 749 with diabetes and 5,320 without diabetes, were analyzed, and for cervical cancer, 7,946 participants were included, 785 of whom had diabetes and 7,161 of whom did not.

Table 1 compares the baseline characteristics of the study participants, all of which were significantly different between people with diabetes and those without. Participants with diabetes were older, included a lower proportion of women, were less educated, had lower income levels, and were less likely to be covered through the NHI program (p< 0.001) than people without diabetes.

Table 2 shows the gastric cancer screening rate in people with and without diabetes. The overall lifetime screening rate for gastric cancer in people with diabetes was lower (53.5%) than in those without diabetes (59.5%, p< 0.001). In a subgroup analysis by age group, gender, income, education, residential area, and type of medical insurance, a significantly lower lifetime screening rate was found in people with diabetes who were 70 years of age or older, women, in the highest income group, less educated (elementary school or less), urban residence, and covered by the NHI program than their counterparts without diabetes in the same socio-demographic groups. In participants 70 years of age or more, the lifetime screening rate was less than 50.0%, irrespective of their diabetes status. The recommended gastric cancer screening rate in people with diabetes was also lower (38.9%) than in those without diabetes (42.9%, p< 0.001). Participants 70 years of age or more with diabetes had a lower recommended screening rate than the same age group without diabetes. In addition, women participants with diabetes and those who lived in urban areas with diabetes had lower recommended screening rates than their counterparts without diabetes.

Table 3 shows the breast cancer screening rate in people with and without diabetes. The overall lifetime screening rate for breast cancer in people with diabetes was significantly lower (60.5%) than in those without diabetes (71.5%, p< 0.001). In the subgroup analyses, significantly lower screening rates were found in individuals with diabetes aged 50-59, with lowest income level, less education (elementary school or less), urban residence, and coverage by the NHI program than in their non-diabetic counterparts. The overall recommended breast cancer screening rate in people with diabetes was lower (38.8%) than in people without diabetes (44.6%, p< 0.001). Moreover, people with diabetes who were aged 60 or more, in the lowest income group, had an educational level of elementary school or less or high school, lived in urban areas, and those who were covered by the NHI program showed significantly lower recommended screening rates than those without diabetes.

Table 4 shows the cervical cancer screening rate in people with and without diabetes. The overall lifetime screening rate for cervical cancer in participants with diabetes was significantly lower (49.1%) than in people without diabetes (59.6%, p< 0.001). In the subgroup analysis, a significantly lower lifetime screening rate was found in people with diabetes aged 40-49 and 50-59, with the lowest educational level (elementary school or less), living in either urban or rural areas, with NHI coverage, and in the lowest or highest income groups than in their counterparts without diabetes in the same socioeconomic groups. The overall recommended cervical cancer screening rate in people with diabetes was lower than in people without diabetes (35.1 vs. 51.2%, p<0.001). In the subgroup analysis, people with diabetes aged 30-39 and 50-59, with the lowest and highest income levels, with middle-school and high-school educations, who lived in either rural or urban areas, and who were covered by NHI showed significantly lower screening rates than those without diabetes in the same socioeconomic groups.

Table 5 shows multivariate logistic regression results for gastric, breast, and cervical cancer screening in diabetic and non-diabetic participants after adjusting for age, gender, income level, education, residential area, and health insurance. People with diabetes showed significantly lower lifetime screening rates for gastric and cervical cancer (odds ratio [OR], 0.8; 95% CI, 0.7 to 0.9 and OR, 0.7; 95% CI, 0.6 to 0.9), and significantly lower regular screening rates for breast and cervical cancer (OR, 0.7; 95% CI, 0.6 to 0.9 and OR, 0.7; 95% CI, 0.5 to 0.9).

## DISCUSSION

To the best of the authors’ knowledge, this is one of the first studies conducted to evaluate and compare cancer screening rates between a group at high risk for cancer (people with diabetes) and non-high-risk group (non-diabetic participants) in the Korean population. Since the prevalence of diabetes in Korea continues to rapidly increase [14], receiving the appropriate screening for various cancers is important for people with diabetes. However, in this study using nationwide survey and health examination data, the cancer screening rate—including both the lifetime screening rate and the recommended screening rate for gastric, breast, and cervical cancers—were lower in people with diabetes than in people without diabetes, even though in Korea the government provides a NCSP in which screening examinations are provided free or at 10% of their normal charge to reduce the economic burden related to cancer screening.

Previous studies conducted in Western countries have consistently shown lower cancer screening rates in people with diabetes [9,10,16,17], similarly to our study. However, in a more recent study using nationwide surveillance data in the US, women with diabetes showed various screening behaviors compared with those without diabetes, including an equal rate of breast cancer screening, a lower rate of cervical cancer screening, and a higher rate of colorectal cancer screening [8]. However, these studies all targeted women. In this study, for gastric cancer screening, the target population for which includes both men and women, the screening rate in men was not different between the 2 groups, but women with diabetes showed a lower screening rate than women without diabetes, suggesting that women with diabetes were a more vulnerable population regarding the use of screening services, as shown in previous studies [9,10,16,17]. In Korea, invitation letters are sent to the eligible population for each type of cancer screening, and even though people with diabetes generally have more occasions to visit clinics for measurements of glucose levels or obtaining medication, their screening rate was lower. Previous studies have suggested that clinicians may focus on the clinical management of diabetes and its direct complications, while considering the prevention of long-term sequelae to be less important [9,16,17]. However, in Korea, the screening rates for diabetic retinopathy and nephropathy, which are direct complications of diabetes, were also found to be lower [18]. Previous studies have confirmed that this may be due to increased time constraints during screening periods, as many patients wish to receive screening examinations [9,10,19]. Additionally, some studies have proven that physicians who need to see many patients at a time are likely to neglect preventive care in patients with chronic diseases such diabetes, and prefer to focus on complicated cases [20,21]. In addition, this pattern might be caused by a trend for clinical practice in Korea to be focused on treatment rather than prevention. Considering the increasing prevalence of diabetes and the decreasing mortality due to diabetes, long-term management of diabetes using appropriate preventive health care strategies, including cancer screening and addressing other long-term sequelae, should be considered important.

Lower cancer screening rates in older people with diabetes than in people without diabetes in the same age group, especially regarding the recommended screenings for gastric and breast cancer, were observed. A study conducted in the US [22] reported similar results, and suggested that this may have been because subjects did not believe that screenings conferred survival benefits. Diabetic people with the lowest education level received less screening than their non-diabetic counterparts. The results of this study agree with other study findings proposing that lower economic and educational levels are correlated with low compliance with screening recommendations, which suggests that sociocultural barriers and adequate health education may be responsible for the lower screening rate in this subgroup [8,19,23-25]. In this study, the cancer screening rate increased with income and educational level in both groups (people with and without diabetes), but in diabetic individuals, the increment was more prominent for education level than for income. Therefore, to increase the cancer screening rate in people with diabetes, efforts should be focused on less-educated people with diabetes. This is consistent with previous studies showing that higher education was a positive predictor for both tests among diabetic women [10]. Urbanity also was not left out in this study, and we observed a lower screening rate among diabetic individuals living in urban areas than among their non-diabetic urban counterparts. It is challenging to see how this may reflect real-life circumstances, as participants living in urban areas have greater opportunities to receive screenings than their rural counterparts and are aware of the benefits of screening. After adjusting for other socioeconomic factors, diabetic participants still showed lower lifetime screening and recommended screening rates for all 3 cancers than those without diabetes.

Although this study has several limitations, such as the inclusion of self-reported data without matching medical records, we believe that the bias was minimal and that the results are representative of the Korean population. We did not take into consideration the characteristics of diabetes (type and duration), which may influence compliance with screening guidelines, and we likewise did not have information that could help measure the efficacy of diabetes care management programs. In addition, we were not able to estimate colorectal and liver cancer screening rates, even though they are included in the NCSP. Both of these cancers have more than one screening method. For liver cancer screening, an alpha-fetoprotein test and sonography are recommended, and for colorectal cancer, a yearly fecal occult blood test in combination with colonoscopy or a double-contrast enema in the case of abnormal findings is recommended. We considered liver cancer screening, which targets high-risk populations such as hepatitis B or C carriers and individuals with liver cirrhosis, but it was not considered appropriate to make a comparison between people with and without diabetes in those populations. In addition, for colorectal cancer screening, the options in the questionnaire regarding the screening interval included less than 2, 2-5, 5-10, and 10 years or more, in contrast to the NCSP guidelines. This ambiguity, and the limited choices on the questionnaire, led us not to analyze colorectal cancer in this study. However, screening for liver and colorectal cancer will be a point of concern for future research.

In conclusion, this study showed a lower screening rate in people with diabetes, and we propose that in the future, continuous public health efforts should emphasize the importance of long-term preventive care, including cancer screening, for high-risk populations such as individuals with diabetes. We further suggest that future research should focus on the cancer screening rate by type and duration of diabetes and the medication used.

## Figures and Tables

**Table 1.** Socio-demographic characteristics of the study participants in the KNHANES, 2007-2009

	With diabetes	Without diabetes	p-value
Age (yr)			<0.001
30-39	79 (5.2)	3,157 (25.9)	
40-49	198 (12.9)	2,967 (24.3)	
50-59	324 (21.6)	2,363 (19.3)	
60-69	507 (33.8)	2,005 (16.4)	
70+	397 (26.5)	1,702 (13.9)	
Gender			<0.001
Men	727 (48.5)	5,114 (41.9)	
Women	773 (51.5)	7,080 (58.1)	
Income			<0.001
Lowest	509 (33.9)	2,433 (19.9)	
Lower	405 (27.0)	2,890 (23.7)	
Higher	287 (19.1)	3,268 (26.8)	
Highest	252 (16.8)	3,324 (27.3)	
Missing	47 (3.1)	279 (2.3)	
Education			
Elementary school	761 (50.7)	3,652 (29.9)	
Middle school	228 (15.2)	1,503 (12.3)	<0.001
High school	319 (21.2)	3,932 (32.3)	
College or more	181 (12.1)	3,074 (25.2)	
Missing	11 (0.7)	33 (0.3)	
Residential area			0.12
Urban	1,051 (70.1)	8,777 (71.9)	
Rural	449 (29.9)	3,417 (28.1)	
Health insurance			<0.001
NHI	1,372 (91.4)	11,676 (95.7)	
MAP	112 (7.5)	416 (3.4)	
No health insurance	14 (0.9)	101 (0.8)	
Missing	2 (0.1)	1 (0.1)	
Diabetes diagnosis			
Diagnosed by a physician	1,145 (76.3)		
Never diagnosed	355 (23.7)		
Diabetes treatment			
Ever treated	958 (63.8)		
Never treated	542 (36.1)		

Values are presented as number (%). KNHANES, Korea National Health and Nutrition Examination Survey; NHI, National Health Insurance; MAP, Medical Aid program.

**Table 2.** Gastric cancer screening rate in diabetic and non-diabetic participants (ever screened and recommended screening)

	Ever screened			Recommended screening		
	Diabetes (95% CI)	No diabetes (95% CI)	p-value	Diabetes (95% CI)	No diabetes (95% CI)	p-value
Total	53.5 (50.1, 59.9)	59.5 (58.2, 60.8)	<0.001	38.9 (35.7, 42.2)	42.9 (41.6, 44.2)	<0.001
Age (yr)						
40-49	55.2 (46.9, 59.7)	57.5 (55.4, 59.7)	0.59	40.2 (32.4, 48.5)	41.9 (39.9, 44.1)	0.68
50-59	60.4 (53.4, 67.1)	65.8 (63.4, 68.2)	0.14	46.9 (40.2, 53.7)	47.8 (45.4, 50.3)	0.80
60-69	59.5 (53.9, 64.9)	64.3 (61.5, 66.9)	0.13	42.5 (37.0, 48.1)	46.5 (43.7, 49.2)	0.21
70+	33.7 (28.6, 39.3)	45.3 (42.4, 48.3)	<0.001	21.4 (17.3, 26.1)	30.1 (27.6, 32.7)	0.002
Gender						
Men	55.5 (50.8, 60.5)	58.5 (56.5, 60.5)	0.28	41.6 (36.9, 46.4)	42.6 (40.7, 44.6)	0.69
Women	50.8 (46.4, 55.5)	60.3 (58.6, 61.9)	<0.001	35.5 (31.3, 39.9)	43.1 (41.4, 44.7)	0.002
Income						
Lowest	47.2 (41.9, 52.6)	51.9 (49.4, 54.6)	0.12	31.9 (27.1, 37.3)	37.0 (34.5, 39.6)	0.09
Lower	54.8 (48.3, 61.3)	56.6 (53.9, 59.3)	0.63	39.1 (32.9, 45.6)	39.4 (36.8, 42.1)	0.91
Higher	54.8 (46.7, 62.7)	58.7 (56.1, 61.3)	0.36	41.9 (34.5, 49.6)	41.8 (39.3, 44.4)	0.99
Highest	59.9 (51.9, 67.4)	68.6 (66.2, 71.1)	0.03	45.6 (37.8, 53.5)	51.6 (49.1, 54.2)	0.16
Education						
Elementary school	48.1 (43.5, 52.8)	54.9 (52.8, 56.8)	0.009	35.1 (30.7, 39.7)	38.8 (36.8, 40.8)	0.15
Middle school	54.7 (46.1, 63.0)	62.6 (59.5, 65.7)	0.08	40.3 (32.5, 48.6)	44.1 (40.8, 47.3)	0.41
High school	59.4 (52.2, 66.3)	58.1 (55.6, 60.5)	0.72	43.1 (36.1, 50.3)	41.1 (38.7, 43.5)	0.60
College or more	61.5 (51.4, 70.8)	66.5 (63.6, 69.4)	0.33	45.1 (35.6, 55.1)	51.5 (48.4, 54.5)	0.23
Residential area						
Urban	52.4 (48.5, 56.4)	60.0 (58.5, 61.5)	<0.001	37.7 (33.9, 41.6)	43.1 (41.5, 44.5)	0.01
Rural	56.9 (51.4, 62.5)	57.7 (55.4, 60.1)	0.80	42.6 (37.1, 48.4)	42.2 (39.9, 44.5)	0.90
Health insurance						
NHI	54.6 (51.3, 58.1)	60.1 (58.8, 61.4)	0.004	39.6 (36.2, 43.1)	43.1 (41.9, 44.5)	0.05
MAP	40.0 (29.2, 51.9)	48.2 (41.9, 54.7)	0.22	30.1 (19.9, 42.6)	37.8 (31.7, 44.3)	0.26

CI, confidence interval; NHI, National Health Insurance; MAP, Medical Aid program.

**Table 3.** Breast cancer screening rate in diabetic and non-diabetic participants (ever screened and recommended screening)

	Ever screened			Recommended screening		
	Diabetes (95% CI)	No diabetes (95% CI)	p-value	Diabetes (95% CI)	No diabetes (95% CI)	p-value
Total	60.5 (57.7, 64.8)	71.5 (69.9, 73.1)	<0.001	38.8 (34.5, 43.3)	44.6 (42.9, 46.5)	<0.001
Age (yr)						
40-49	69.1 (55.4, 80.1)	72.7 (70.1, 75.3)	0.56	54.3 (40.8, 67.1)	52.9 (50.1, 55.8)	0.85
50-59	73.1 (63.3, 81.1)	83.6 (81.1, 85.8)	0.01	51.6 (41.6, 61.4)	59.8 (56.6, 62.9)	0.12
60-69	67.9 (60.3, 74.5)	72.8 (69.4, 76.2)	0.20	38.8 (32.1, 46.1)	48.7 (45.1, 52.4)	0.02
70+	39.6 (32.7, 46.9)	43.2 (39.4, 47.4)	0.40	20.1 (15.4, 25.8)	27.9 (24.8, 31.4)	0.02
Income						
Lowest	52.7 (45.7, 59.6)	62.5 (59.3, 65.7)	0.01	30.4 (24.5, 36.7)	43.7 (40.5, 47.1)	<0.001
Lower	62.2 (53.6, 70.2)	68.5 (65.2, 71.7)	0.15	41.9 (33.5, 50.7)	46.1 (42.6, 49.5)	0.39
Higher	65.2 (53.1, 75.6)	72.9 (69.5, 76.2)	0.18	41.7 (30.6, 53.7)	50.9 (47.4, 54.4)	0.14
Highest	76.5 (65.4, 84.8)	81.9 (79.1, 84.4)	0.25	49.4 (38.2, 60.7)	60.9 (57.4, 64.3)	0.05
Education						
Elementary school	56.5 (51.1, 61.8)	63.8 (61.4, 66.2)	0.01	35.1 (30.1, 40.4)	43.8 (41.4, 46.3)	0.003
Middle school	68.5 (54.7, 79.6)	74.9 (70.7, 78.7)	0.31	44.9 (31.6, 58.9)	51.1 (46.7, 55.5)	0.41
High school	70.3 (57.4, 80.5)	75.5 (72.6, 78.2)	0.37	42.6 (31.7, 54.2)	54.8 (51.5, 58.1)	0.05
College or more	78.1 (52.1, 92.1)	82.5 (78.4, 86.1)	0.64	74.1 (49.4, 89.4)	61.5 (56.5, 66.1)	0.27
Residential						
Urban	59.8 (54.3, 65.1)	72.2 (70.3, 74.1)	<0.001	37.3 (32.2, 42.7)	50.7 (48.7, 52.7)	<0.001
Rural	62.8 (55.1, 70.1)	69.2 (66.4, 71.9)	0.11	41.7 (34.5, 49.4)	49.8 (46.8, 52.7)	0.05
Medical insurance						
NHI	61.8 (57.1, 66.5)	72.4 (70.8, 73.9)	<0.001	39.1 (34.5, 43.9)	51.4 (49.6, 53.1)	<0.001
MAP	47.4 (34.1, 61.1)	56.6 (49.1, 63.8)	0.26	32.8 (21.4, 46.7)	36.6 (29.7, 44.1)	0.63

CI, confidence interval; NHI, National Health Insurance; MAP, Medical Aid program.

**Table 4.** Cervical cancer screening rate in diabetic and non-diabetic participants (ever screened and recommended screening)

	Ever screened			Recommended screening		
	Diabetes (95% CI)	No diabetes (95% CI)	p-value	Diabetes (95% CI)	No diabetes (95% CI)	p-value
Total	49.1 (44.7, 53.5)	59.6 (58.2, 61.0)	<0.001	35.1 (31.0, 39.4)	51.2 (49.7, 52.6)	<0.001
Age (yr)						
30-39	54.1 (36.6, 70.4)	57.1 (54.4, 59.8)	0.73	27.7 (15.2, 44.8)	51.2 (48.3, 53.8)	0.007
40-49	48.2 (35.1, 61.6)	64.9 (62.2, 67.5)	0.01	56.9 (43.5, 69.3)	62.2 (59.4, 65.0)	0.43
50-59	57.3 (47.3, 66.6)	68.3 (65.4, 71.1)	0.03	47.1 (37.3, 69.3)	58.0 (54.8, 61.1)	0.04
60-69	55.2 (47.5, 62.2)	60.3 (56.6, 63.8)	0.23	33.8 (27.3, 41.1)	41.3 (37.8, 44.9)	0.07
70+	36.9 (30.1, 44.3)	34.7 (31.2, 38.4)	0.58	18.2 (13.6, 24.0)	20.1 (17.2, 23.3)	0.55
Income						
Lowest	40.6 (34.1, 47.5)	49.8 (46.6, 52.9)	0.02	22.3 (17.5, 27.5)	37.3 (34.3, 40.4)	<0.001
Lower	56.3 (48.1, 64.2)	58.9 (56.1, 61.7)	0.55	41.5 (33.4, 50.1)	47.8 (44.9, 50.8)	0.17
Higher	56.4 (45.2, 67.1)	61.4 (58.7, 64.0)	0.39	42.4 (31.6, 54.0)	52.3 (49.5, 55.0)	0.10
Highest	51.3 (40.5, 62.1)	65.1 (62.6, 67.6)	0.01	44.9 (34.5, 55.8)	62.7 (59.9, 65.4)	0.001
Education						
Elementary school	46.9 (41.5, 52.4)	53.8 (51.4, 56.3)	0.02	32.2 (27.3, 37.7)	37.8 (35.4, 40.7)	0.06
Middle school	58.5 (45.2, 70.2)	64.2 (60.3, 67.9)	0.40	38.6 (26.6, 52.3)	53.1 (48.8, 57.3)	0.04
High school	51.2 (40.8, 61.5)	61.3 (59.1, 63.7)	0.06	39.5 (29.9, 49.9)	56.6 (54.1, 59.1)	0.001
College or more	55.8 (37.0, 73.1)	61.9 (58.9, 64.8)	0.52	54.2 (35.7, 71.7)	59.1 (55.9, 62.1)	0.62
Residential						
Urban	49.8 (44.6, 55.0)	60.2 (58.8, 61.6)	<0.001	35.5 (30.7, 40.7)	53.2 (51.5, 54.8)	<0.001
Rural	46.7 (39.2, 54.2)	47.7 (40.6, 54.9)	0.04	35.5 (30.7, 40.7)	53.2 (51.5, 54.8)	<0.001
Medical Insurance						
NHI	49.3 (44.1, 54.1)	81.2 (80.1, 82.5)	<0.001	35.5 (31.2, 40.1)	52.2 (50.7, 53.6)	<0.001
MAP	52.4 (38.8, 65.6)	60.1 (52.4, 67.3)	0.56	33.1 (26.3, 38.1)	31.6 (24.4, 38.6)	0.83

CI, confidence interval; NHI, National Health Insurance; MAP, Medical Aid program.

**Table 5.** Multivariate logistic regression^1^ in diabetic and non-diabetic participants

		Ever screened		Recommended screening	
		OR (95% CI)	p-value	OR (95% CI)	p-value
Gastric cancer	No	1.0 (reference)		1.0 (reference)	
	Yes	0.8 (0.7,0.9)	0.02	0.9 (0.7,1.0)	0.19
Breast cancer	No	1.0 (reference)		1.0 (reference)	
	Yes	0.8 (0.6,1.0)	0.06	0.7 (0.6, 0.9)	0.02
Cervical cancer	No	1.0 (reference)		1.0 (reference)	
	Yes	0.7 (0.6, 0.9)	0.01	0.7 (0.5, 0.9)	0.004

OR, odds ratio; CI, confidence interval.

1 Adjusted for age, gender, income level, education, residential area, and medical insurance.

## Appendix 1. Cancer screening protocol issued by the national screening program in Korea

**Table:**

Cancer type	Target population	Screening method	Interval (yr)
Stomach cancer	≥40 yr old	Gastric endoscopy or upper gastro-intestinal series	Every 2
	Men and women		
Colorectal cancer	≥50 yr old	Fecal occult blood test and if positive, colonoscopy or barium Enema	Every
	Men and women		
Breast cancer	≥40 yr old	Mammography	Every 2
	Women		
Cervical cancer	≥30 yr old	Pap smear	Every 2
	Women		
